# Asking for help: the development of a simulation-based mental health application to enhance depression literacy, mental health communication, and help-seeking among Black autistic youth

**DOI:** 10.3389/fpsyt.2026.1766641

**Published:** 2026-04-01

**Authors:** Ed-Dee G. Williams, Fatima Vakil, Oluwatobi Abubakare, Angelina Latin, Lauren Ware, David Nelson, Matthew J. Smith

**Affiliations:** 1 School of Social Work, Boston College, Chestnut Hill, MA, United States; 2 Department of Psychology, Boston College, Chestnut Hill, MA, United States; 3 School of Social Work, University of Michigan, Ann Arbor, MI, United States

**Keywords:** autism, Black/African American, depression, help-seeking, mental health intervention, mental health literacy (MHL)

## Abstract

Black autistic youth experience disproportionately high rates of depression and face intersecting barriers such as racial discrimination, stigma, and limited access to care, yet few interventions address their needs. This study introduces Asking for Help (A4H), a culturally responsive, simulation-based intervention designed to improve depression literacy and help-seeking skills through an e-learning module and interactive conversation practice. Guided by mental health literacy theory, the Theory of Help-Seeking Behavior, the Theory of Planned Behavior, and Disability Critical Theory, A4H was developed using community-engaged and user-centered design principles. Usability testing employed a mixed-methods design with 32 participants (12 youth, 10 caregivers, 8 specialists) using the System Usability Scale (SUS), Patient Health Questionnaire-9 (PHQ-9), and semi-structured interviews. Black autistic youth reported moderate depressive symptoms (mean PHQ-9 = 14.7) and rated usability slightly below benchmark (mean SUS = 66.2), while caregivers and specialists scored higher (73.5 and 71.0). Qualitative feedback highlighted cultural relevance and immediate feedback as strengths, with recommendations for simplified language, improved navigation, and multimodal supports; emotional safety and trust were critical for engagement. No short-term symptom change was observed, consistent with the formative design. Findings indicate A4H is feasible and culturally responsive but requires refinements before efficacy testing to assess impacts on literacy, help-seeking intentions, and communication skills.

## Introduction

A recent call to action by Black autism scholars, advocates, and practitioners (1) emphasized the urgent need for increased support and services tailored to Black autistic youth. They argue that the rising rates of autism diagnoses among Black youth demand a stronger focus on culturally responsive and racially specific interventions that address their unique needs. The Asking for Help (A4H) simulation program was developed, in part, as a direct response to this call and from a broader recognition of the lack of mental health supports specifically designed for Black autistic youth.

A4H is a culturally informed, race-specific virtual mental health simulation tool created to help Black autistic youth expand their understanding of depression and build skills for effectively seeking support for depressive symptoms. The program was developed using a community-engaged research approach and a user-centered design process. Its content and interface were co-designed with members of the Black autism community and informed by data collected through interviews and surveys with Black autistic youth, their parents and caregivers, and community advocates, as well as ongoing input from a community advisory board.

A4H consists of two main components: (1) an e-learning module that provides foundational depression literacy training to users, and (2) a simulated conversation in which the user practices communicating depressive symptoms to a trusted adult, in the current iteration is a special education teacher named Mrs. Daniels. The program’s ongoing development is guided by iterative feasibility and usability testing with Black autistic youth, their families, autism care specialists, and mental health professionals.

This study outlines the theoretical framework guiding the intervention, describes its initial design, presents pilot-phase participant feedback, details refinements made in response to that feedback, and discusses future directions for evaluating A4H’s effectiveness and implementation.

Autism is a neurodevelopmental condition characterized by differences in social communication and interaction, along with restricted, repetitive behaviors and atypical sensory processing (2, 3). Clinically, it is understood as a spectrum encompassing a wide range of presentations and varying levels of support needs (4, 5). Beyond its clinical definition, autism also functions as a social identity shaped by shared lived experiences and societal perceptions; autistic individuals often gain psychological benefits from strong group identity and community solidarity (6, 7), yet they also face stigma, masking pressures, and minority stress when navigating environments designed for non-autistic norms (8, 9). Research consistently shows elevated rates of co-occurring mental health conditions among autistic individuals, such as anxiety, depression, ADHD, PTSD, OCD, eating disorders, and substance use disorders, stemming from both neurodevelopmental factors and systemic ableism (3, 10, 11).

Recent data indicate a notable surge in autism diagnoses among Black children, a shift reflecting both improved detection and a narrowing of historic disparities. CDC surveillance via the Autism and Developmental Disabilities Monitoring (ADDM) network shows autism prevalence rising from 1 in 36 children in 2020 to 1 in 31 by 2022, with Black children now exhibiting the highest rates in many regions (e.g., 1 in 27 in Maryland compared to 1 in 52 for white peers) (12, 13). Data from JAMA Open Network (2011–2022) further indicate that racial and ethnic minority groups, including Black youth, have experienced relatively greater increases in diagnosis rates over the last decade.

## Autism and depression

Depression is a common co-occurring condition among autistic youth, affecting approximately 25% of autistic youth compared to 8–12% of their non-autistic peers (14–16). This elevated prevalence is linked to multiple risk factors, including core social communication challenges that hinder the ability to express mental and emotional needs (17). Additionally, experiences of stigma, bullying, and social exclusion in an ableist society further increase vulnerability to depression and anxiety (18). Youth with untreated depression face heightened risks for suicide, self-harm, and substance abuse, and the suicide rate among autistic youth is estimated to be nearly ten times higher than the national average (19, 20). Although approximately 55% of autistic youth receive psychiatric medications for mood disorders (21, 22), there remains a critical gap in interventions that equip them with skills to engage in help-seeking conversations which could also decrease untreated depression.

These concerns are even more pronounced for Black autistic youth, who face the compounded effects of racism, ableism, and cultural stigma surrounding mental health. Despite the growing recognition of elevated suicide risk among autistic youth, there is a lack of research and targeted supports that address the unique mental health needs of Black autistic individuals. Black youth broadly often report comparable or higher rates of depressive symptoms than their White peers yet are less likely to receive a formal depression diagnosis or treatment (23–25). Structural racism, particularly racial discrimination, is a significant risk factor, with frequent exposure strongly associated with depressive symptoms across adolescence (26, 27). Evidence from longitudinal studies demonstrates that racial discrimination predicts increases in depressive symptoms over time, underscoring its role as a persistent psychosocial stressor (27). In addition to systemic barriers, stigma surrounding mental health within some Black communities further compounds these disparities. Cultural norms that discourage disclosure of emotional distress, fear of being labeled, and mistrust of mental health systems can contribute to lower help-seeking behaviors and reduced service utilization in Black communities (28, 29). The intersection of these identities, Black autistic youth, remains critically understudied. Emerging research suggests complex dynamics: for example, Black autistic youth report less racial discrimination than their non-autistic Black peers, attributing discrimination more to autism than race (30). Yet, Black autistic youth appear more likely to experience depressive symptoms than White autistic youth, possibly due to the compounded effects of racism and ableism (31). This underscores the urgent need for culturally grounded, identity-affirming interventions that promote depression literacy and empower Black autistic youth to seek help safely and effectively.

## Rationale for A4H development

Black autistic youth face a distinct set of challenges that significantly increase their risk for depression and other mental health concerns. These challenges stem from the intersection of systemic racism, socioeconomic disadvantage, and the social-emotional and communicative differences associated with autism (32, 33). In this context, communicative differences refer to well−documented autistic communication patterns such as more direct or formal language use, difficulties interpreting or using nonverbal cues (e.g., gaze, facial expressions, or gestures), challenges with conversational turn−taking, and more literal interpretation of language, all of which can contribute to social misunderstanding and marginalization (34, 35). Black youth are disproportionately exposed to under-resourced schools, limited access to culturally affirming mental health care, and chronic stressors related to racial discrimination. When these structural inequities intersect with autism-related vulnerabilities, such as peer rejection, bullying, and social isolation, Black autistic youth emerge as a critically underserved and at-risk population (32, 36).

Simulation-based and virtual reality (VR) interventions have shown promise in supporting developmental and mental health outcomes among autistic youth. A systematic review by Yang et al. (37) found that VR interventions significantly improve social skills, emotional recognition, and behavioral regulation in children and adolescents with autism spectrum disorder (ASD). Similarly, Zhao et al. (38) demonstrated that VR-based tools enhance cognitive flexibility and social communication among autistic children. These findings are echoed by Williams et al. (39), who documented the growing use of simulation-based approaches in mental health education, particularly for increasing access and engagement among youth populations.

Despite these technological advances, culturally tailored mental health interventions remain scarce, especially for Black autistic youth. Research consistently shows that culturally responsive interventions are more effective than generic approaches in improving mental health outcomes for Black youth (40, 41). Cultural responsiveness, for the purposes of this study, refers to the practice of designing, delivering, and evaluating interventions in ways that meaningfully reflect, respect, and adapt to the unique cultural identities, values, communication styles, lived experiences, and contextual realities of the heterogeneous Black autistic community (42, 43). Within autism research, there is increasing recognition of the lack of racial and ethnic representation in intervention design and evaluation. Interventions explicitly developed for Black autistic individuals and their families, such as the Best FACES Forward advocacy program, have demonstrated positive outcomes in empowerment, communication, and perceived advocacy ability (44).

To address these gaps, an intersectional and sociocultural framework grounded in Disability Critical Theory (DisCrit) is essential (45). The continued reliance on universal interventions, often developed and validated using predominantly White samples, reinforces the assumption that White youth represent the normative standard. This paradigm marginalizes the lived experiences of Black autistic youth and undermines the relevance and effectiveness of mental health supports (46).

In response to the growing call for culturally adapted interventions that address the specific social, emotional, and mental health needs of autistic individuals from racially marginalized backgrounds, researchers and practitioners have increasingly focused on enhancing social communication, promoting emotional well-being, and supporting caregivers (42, 47). Within this context, simulation-based interventions explicitly tailored for Black autistic youth represent a promising avenue for integrating culturally responsive practices with evidence-based, technology-driven tools. Such interventions not only aim to improve developmental and mental health outcomes but also affirm racial and neurodivergent identities, contributing to more equitable and affirming systems of care.

## Description of A4H

**Figure 1:**
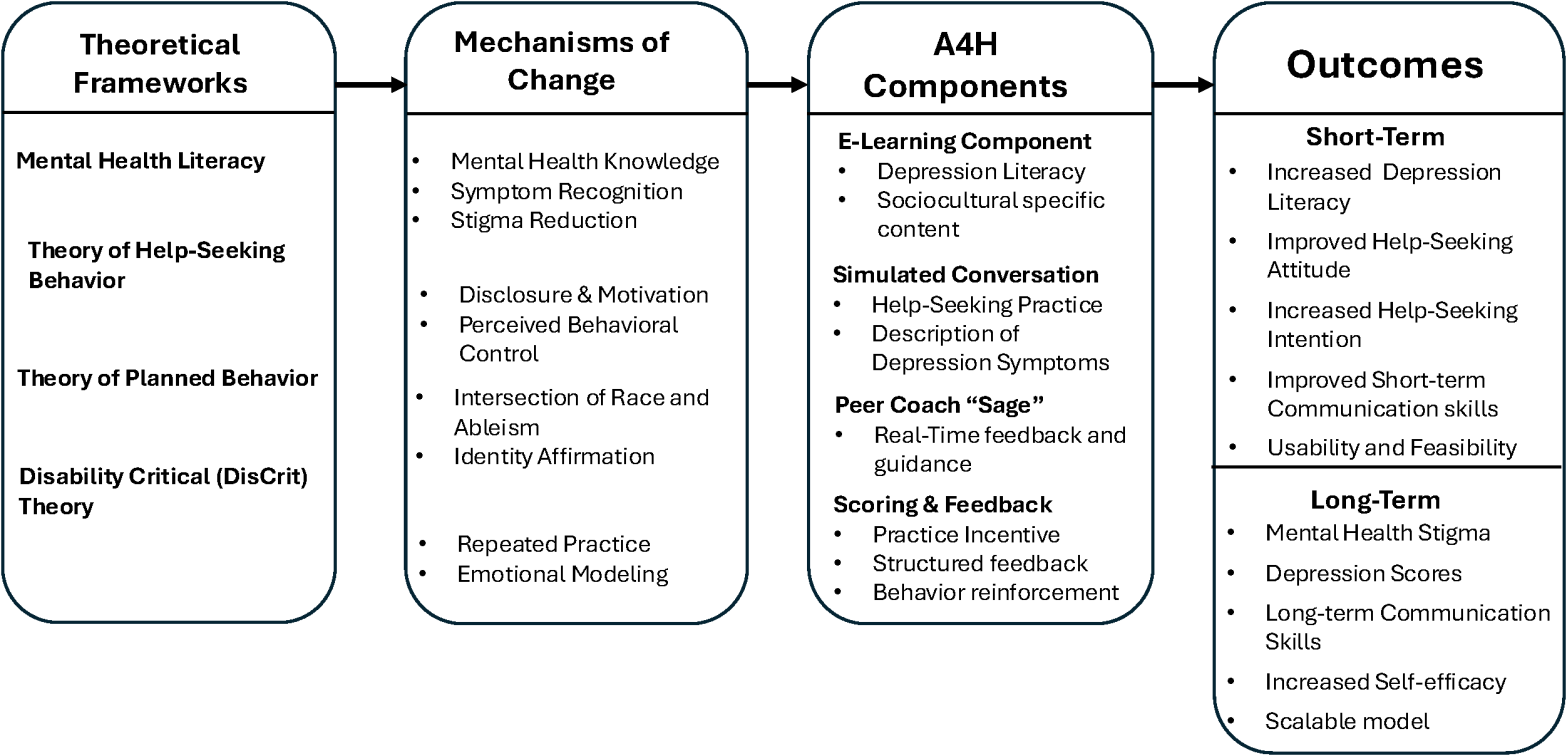
Logic model for Asking for Help (A4H): Theories, mechanisms of change, components, and expected outcomes. This logic model illustrates the theoretical foundation, mechanisms of change, core components, and anticipated outcomes of *Asking for Help* (A4H), a culturally responsive, simulation-based intervention for Black autistic youth. Foundational theories include Mental Health Literacy, the Theory of Help-Seeking Behavior, the Theory of Planned Behavior, and Disability Critical Theory (DisCrit). Mechanisms of change target depression literacy, stigma reduction, self-efficacy, communication skills, and intersecting identitjes of race and ableism. Core components comprise an e-learning module, interactive simulation with a virtual teacher, real-time peer coaching, and a scoring/feedback system. Outcomes are organized by timeframe: short-term (e.g., improved depression literacy, help-seeking intentions), and long-term (e.g., reduced depressive symptoms, improved mental health trajectories Adapted from the A4H development framework described in Williams et al. (48).

A4H is a simulation-based mental health literacy intervention designed to address the unique and intersecting challenges experienced by Black autistic youth. Grounded in culturally affirming and developmentally appropriate content, the program seeks to improve mental health-related outcomes by strengthening knowledge of depression, mental health communication skills, and help-seeking behaviors. Its evidence base integrates four complementary theoretical frameworks, mental health literacy, the theory of help-seeking behavior, theory of planned behavior, and Disability Critical Theory, into two core components: (1) an initial e-learning module focused on depression literacy, and (2) an interactive simulation in which participants engage in a conversation with a virtual special education teacher to practice effectively describing depressive symptoms. Together, these components aim to empower Black autistic youth with the skills and confidence needed to navigate mental health challenges and seek appropriate support (see **Figure 1**).

Black autistic youth face compounded barriers to early recognition and support for depression, including diagnostic overshadowing of mood symptoms by autism−related differences (49), racialized and disability−based stigma, and reduced access to culturally responsive care (26). These challenges are further intensified by culturally patterned expressions of depression within African American communities; as Walton and Payne (50) demonstrates, depressive symptoms among Black individuals often manifest through irritability, somatic complaints, or high−functioning perseverance rather than prototypical affective markers. Such patterns increase the likelihood that depressive symptoms in Black autistic youth will be misinterpreted as behavioral concerns or attributed solely to autism, impeding accurate identification by both youth and adults and making help−seeking feel risky or ineffective (51). Targeting depression literacy (to improve recognition and reduce stigma) and help−seeking skills (to increase communication confidence, planning, and navigation) directly addresses modifiable barriers that are proximal to service connection in this population.

We employed evidence-based, community-engaged methods (52, 53) and a user-centered approach (54, 55) to develop *Asking for Help (A4H)* in collaboration with SIMmersion LLC, a company specializing in immersive, interactive training programs that use virtual human role-players to support skill development through realistic conversations. Together, we created a prototype that is currently undergoing feasibility, usability, and adaptability testing. The development process was iterative, incorporating multiple rounds of review and refinement based on feedback from both the design team and the Community Advisory Board (CAB) to ensure cultural relevance, clarity, and authenticity.

## Community engaged research and user-centered design

Community-engaged research is an approach to research that actively involves community members, organizations, and other stakeholders in the research process. Community engagement, as defined by the Clinical and Translational Science Awards (CTSA) Consortium, is a “continuum of community involvement,” meaning it can be measured by the degree of a community’s active involvement in the research process (56, 57). The CTSA describes nine key tenets to community engagement, (1) clearly define the purpose, goals, and target population for community change; (2) gain comprehensive knowledge of the community; (3) build relationships through active interaction; (4) support community self-determination; (5) establish partnerships with the community; (6) honor cultural diversity and values; (7) leverage community assets and strengthen capacity; (8) remain adaptable; and (9) commit to sustained, long-term collaboration.

User-centered design (UCD) is a methodological approach that foregrounds the needs, preferences, and lived experiences of end users throughout the research and intervention development process (54). Rooted in the discipline of computer engineering, UCD emphasizes the creation of systems and programs that are not only functional but also optimized for usability and effectiveness from the perspective of the intended users (58, 59). Theoretically, UCD draws on principles of participatory design and human-computer interaction, which advocate for iterative, collaborative processes that integrate user feedback at every stage. In mental health research, UCD is particularly relevant as it ensures that interventions are culturally responsive, accessible, and aligned with the real-world contexts and challenges faced by diverse populations, thereby enhancing engagement and treatment outcomes (60). The development of A4H was guided by the core principles of User-Centered Design (UCD) as outlined in the ISO 9241–210 standard (61). These principles emphasize grounding design decisions in a deep understanding of users’ needs, goals, and contexts; involving users throughout the design and implementation process; employing iterative cycles of prototyping and refinement informed by user feedback; and prioritizing usability and accessibility to ensure inclusivity. Applying these principles was critical for A4H because the platform aims to address mental health disparities among Black autistic youth, a population that experiences compounded marginalization due to intersecting identities. By centering the voices and lived experiences of these youth and their families, the design process sought to create a resource that is culturally responsive, developmentally appropriate, and accessible for diverse abilities. This approach not only enhances usability but also advances equity by ensuring that interventions are tailored to the unique sociocultural and neurodiverse contexts of the intended users.

CER provided a framework for building trust and sustained partnerships with community stakeholders, honoring cultural values, and leveraging community assets to guide decision-making (57, 61). At the same time, UCD emphasized iterative design grounded in a deep understanding of user needs, active involvement of youth and caregivers throughout the process, and prioritization of usability and accessibility (54, 61). Integrating these approaches, A4H was shaped through continuous feedback loops that centered lived experiences and sociocultural contexts, ensuring the platform not only meets functional requirements but also advances mental health equity for a population facing intersecting forms of marginalization. To further promote equity and accessibility, A4H is delivered as a mobile health application, recognizing that low-income families often rely on smartphones as their primary means of internet access. Mobile optimization reduces technology-related barriers, enabling participants to engage with the program in familiar and flexible settings, whether at home, school, or on the go, while enhancing usability and completion rates. This mobile-first strategy also supports scalability and broader dissemination by eliminating the need for specialized hardware, positioning A4H within a practical, widely adopted approach to improving mental health literacy and help-seeking behaviors among youth.

## E-learning: depression literacy

The e−learning focused on depression literacy, building upon the foundational principle of the mental health literacy framework that improving individuals’ understanding of mental health and a particular mental health condition promotes early recognition, appropriate help−seeking, and effective self−management (62, 63). The mental health literacy framework emphasizes the importance of knowledge and beliefs about mental disorders to promote early recognition, appropriate help−seeking, and effective self−management. Core components include the ability to identify symptoms, understand risk factors and causes, recognize available treatments, and reduce stigma that often prevents individuals from seeking care (63, 64). Strengthening depression literacy is especially critical for Black autistic youth, as culturally patterned expressions of depression within African American communities, such as irritability, somatic complaints, or high−functioning perseverance, may diverge from prototypical diagnostic expectations (50), increasing the likelihood that symptoms are overlooked or misattributed to autism. By improving mental health literacy, A4H looks to empower Black autistic youth to respond effectively to mental health challenges and more effectively seek support.

Furthermore, the e−learning module provides foundational information about depression tailored specifically for Black autistic youth. Guided by key tenets of Disability Critical (DisCrit) theory (45), including the interdependence of racism and ableism and the social construction of race and disability, the module situates depression within the lived realities of Black autistic youth. It highlights how intersecting experiences of racial and disability-based discrimination, bullying, stigma, and systemic inequities compound vulnerability to depression (65). By framing mental health within these structural contexts, A4H helps participants understand that mental health disparities are not solely individual challenges but are deeply connected to systemic oppression, fostering both awareness and empowerment. This structural framing is a key component of A4H’s cultural responsiveness, as it affirms youth’s lived experiences, validates the sociocultural forces shaping their mental health, and counters deficit-oriented narratives that overlook systemic contributors to distress. The content is delivered through text and pre−recorded videos narrated by a simulated peer character, creating a relatable and engaging learning experience.

The session introduces common symptoms of depression, emphasizing those frequently reported by Black autistic individuals, such as persistent sadness, irritability, fatigue, social withdrawal, difficulty concentrating, and somatic complaints (e.g., headaches, stomachaches). These symptoms were identified through formative interviews and supported by prior research on depression among Black youth, guiding their inclusion in the A4H pilot (23, 66). Importantly, the content frames these symptoms within culturally responsive and neurodiversity-informed contexts to foster accurate recognition and empathy, ensuring that participants see their experiences reflected in ways that validate both racial and disability identities.

The initial e-learning content was co-developed with the principal investigator, the community advisory board, and SIMmersion scriptwriters to ensure both technical accuracy and cultural responsiveness. Following the initial draft, the Community Advisory Board (CAB) reviewed the script and provided detailed feedback, which was integrated into subsequent revisions. This iterative process, conducted three times, prioritized cultural relevance, clarity, and authenticity. To enhance accessibility, the finalized script was processed through an AI platform that adapted the language to a sixth-grade reading level while preserving its culturally responsive and neurodiversity-informed content. After this adjustment, the CAB conducted a final review before incorporating the script into the A4H e-learning module.

## Simulated conversation

The simulated conversation uses SIMmersion’s patented PeopleSim^®^ technology, a simulation engine that powers highly realistic virtual human role-players for communication training. Each simulated character is powered by a “virtual brain” that governs memory, emotional states, and behavioral responses, enabling dynamic and authentic conversations. Unlike traditional branching scripts, PeopleSim^®^ uses non-linear logic and a conditional response lottery, allowing varied outcomes and unpredictability across sessions. Characters exhibit emotional modeling, adjusting their tone and trust based on user choices, and offer multiple personality profiles to enhance replay value and skill generalization. Immediate feedback is integrated throughout the experience, reinforcing learning and improving communication competence (67).

Theory of Planned Behavior (TPB) and Waller et al.’s (68) Theory of Help−Seeking Behavior (THSB) address both the individual and structural determinants of help−seeking. The TPB emphasizes that help−seeking is shaped by youths’ attitudes toward seeking support, their perceptions of social norms, and their sense of control over navigating services (69, 70). A4H directly targets these components by improving depression literacy, reducing stigma, and building concrete skills that enhance perceived behavioral control. The THSB adds a critical layer by demonstrating that help−seeking among marginalized groups is profoundly shaped by sociocultural context, historical mistrust, and prior harmful encounters with providers. By combining TPB’s focus on intention formation with THSB’s attention to structural barriers and agency, A4H equips Black autistic youth with the knowledge, confidence, and contextual awareness needed to recognize depression, trust their interpretations of distress, and engage in help−seeking even within systems that have historically marginalized them.

The simulated conversation component of A4H provides participants with a safe, interactive environment to practice communicating depressive symptoms to a trusted adult, a critical step in the help-seeking process. In its current iteration, the simulation features a Black, female-presenting special education teacher named Mrs. Daniels as the conversation partner, ensuring cultural and contextual relevance. Users select one of six commonly reported depressive symptoms among Black autistic youth to focus on during the interaction.

The identity of Mrs. Daniels in the simulation was intentionally chosen in direct response to early feedback from our Community Advisory Board, who emphasized the importance of cultural responsiveness and meaningful representation within the platform’s characters. Reflecting this guidance, Mrs. Daniels was designed as a Black woman whose appearance, including her natural hairstyle, was selected to reflect common cultural expressions within the communities A4H aims to serve. This design choice was intended to enhance the authenticity of the simulation, increase user comfort and relatability, and counter the historical underrepresentation of culturally diverse caregivers in digital mental health tools. Additionally, a virtual “peer coach”, currently named Sage provides real-time verbal and nonverbal feedback, guiding participants toward clearer, more confident responses. This component operationalizes key constructs from the THSB by reducing barriers to disclosure, building communication skills, and normalizing help-seeking as a proactive strategy (68). It also aligns with the TPB by targeting attitudes toward help-seeking, reinforcing supportive norms through culturally affirming content, and enhancing perceived behavioral control through repeated, feedback-informed practice (71, 72). Together, these features aim to strengthen intention and ability to seek mental health support, fostering empowerment and self-advocacy.

## Scoring system and theoretical alignment

A4H incorporates a scoring system designed to provide immediate, structured feedback on participants’ performance during the simulated help-seeking conversation. After completing the virtual conversation, users receive a composite score ranging from 0 to 100 that reflects their overall success in navigating the interaction with Mrs. Daniels. These scores are calculated based on multiple dimensions, including adequate disclosure and clarity of symptoms, effective descriptions of symptom severity and the emotional impact of their feelings, and the user’s ability to continue and maintain the conversation.

This scoring system is not merely a technical feature; it operationalizes key constructs from the intervention’s theoretical foundation. Self-efficacy, grounded in Bandura’s (73) conception of self-efficacy, is reinforced by the scoring system, which boosts participants’ confidence in their ability to disclose depressive symptoms effectively. Higher scores indicate successful navigation of the conversation and clear symptom communication, which serve as mastery experiences, the most influential source of self-efficacy. Immediate feedback and opportunities for repeated practice further strengthen participants’ belief that they can perform help-seeking behaviors in real-world contexts.

The scoring system also aligns with the Theory of Planned Behavior and the Theory of Help-Seeking Behavior (68, 69), specifically through behavioral intentions that influence attitudes, perceived norms, and perceived behavioral control. Favorable scores and feedback normalize help-seeking as an achievable and socially supported behavior, enhancing participants’ intention to seek help. By demonstrating that help-seeking is both possible and valued, the scoring system fosters motivation and readiness to act.

Additionally, consistent with the Theory of Help-Seeking Behavior (69, 74), score components reflect critical stages of the help-seeking process. For example, clarity of symptom disclosure corresponds to the ability to communicate needs, perceived importance reflects motivation to act, and emotional impact signals recognition of symptom severity. These dimensions collectively support progression from problem recognition to active help-seeking.

## Usability of pilot

After completing several iterative steps to establish a robust pilot version of A4H, the next phase focused on evaluating the platform’s usability and gathering comprehensive user feedback. This process was essential for identifying strengths, uncovering barriers to engagement, and informing refinements that would enhance functionality, cultural responsiveness, and accessibility. By systematically analyzing user experiences and incorporating their insights, the development team aimed to ensure that A4H not only met technical standards but also aligned with the real-world needs and preferences of Black autistic youth and their families.

## Methods

To evaluate A4H’s usability and relevance, we employed a user-centered mixed-methods design that integrated quantitative and qualitative approaches. All participants tested A4H virtually, with their sessions recorded via Zoom to capture real-time engagement and navigation patterns. Before using A4H, youth participants completed a pre-survey that collected demographic information (e.g., age, race, gender) and included standardized measures such as the Patient Health Questionnaire-9 (PHQ−9) for depression screening and a general help-seeking questionnaire. Parent/caregiver and autism specialist participants completed a similar demographic survey, excluding the PHQ−9.

The PHQ−9 was included at pre-test to provide a standardized baseline indicator of depressive symptom severity and to contextualize youths’ engagement with the platform. Importantly, in this early-stage feasibility study, the PHQ−9 was conceptualized primarily as a screening and contextualization tool, not as a clinical outcome measure. Because the single 30−minute session was not intended to deliver an intervention dose sufficient to elicit symptom change, PHQ−9 data at this stage served to document baseline characteristics and support safety monitoring.

Following the pre-survey, participants were given access to A4H and instructed to engage with the program for approximately 30 minutes. This duration was selected to allow participants sufficient time to complete the e−literacy training and experience a full conversation for each depressive symptom included in the platform.

Immediately after the session, participants completed electronic surveys assessing usability metrics such as ease of navigation, clarity of content, and perceived usefulness. To complement these quantitative data, semi-structured interviews were conducted to elicit deeper insights into participants’ experiences, including perceptions of cultural responsiveness, accessibility, and overall relevance to their needs. A mixed−methods approach was chosen to capture both the breadth and depth of user experiences. Quantitative data provided structured measures for systematic comparison, while qualitative interviews offered nuanced perspectives on cultural and contextual factors that influence usability. This methodological triangulation enhanced validity and ensured that refinements to A4H were informed by comprehensive, user-driven insights, aligning with the principles of user-centered design and community-engaged research.

Follow-up surveys included the PHQ−9 (75) and the Systems Usability Scale (SUS) (76) to assess participants’ overall perceptions of A4H’s usability. The SUS consists of 10 items rated on a 5−point Likert scale ranging from 1 (Strongly disagree) to 5 (Strongly agree). Items alternate between positive and negative statements about usability (e.g., “I think that I would like to use this system frequently” vs. “I found the system unnecessarily complex”). The PHQ−9 was excluded for parent/caregiver and autism specialist participants.

Although PHQ−9 items were included for Black autistic youth participants in the follow-up survey, this was done to assess the feasibility of administering clinical measures within the study workflow and to ensure no unintended adverse changes occurred following platform use. The study was not designed or powered to detect clinical change, and consistent with its formative focus, PHQ−9 scores are not analyzed or interpreted as symptom-related outcomes in this manuscript.

Finally, because this phase prioritized usability, feasibility, and user experience, we intentionally did not administer quantitative measures of mental health literacy, communication confidence, or self−efficacy. While these constructs are central to the proposed mechanisms of A4H, they are planned for subsequent evaluation in a later pilot trial designed to test proximal and distal outcomes. In this initial phase, minimizing participant burden and emphasizing qualitative and usability-focused metrics were critical to establishing foundational feasibility.

The qualitative interviews were designed to gather context-rich feedback on participants’ initial perceptions and experiences using A4H. Each interview followed a semi-structured protocol that included questions aimed at understanding (1) whether participants agreed with the program’s goals; (2) any problems or difficulties encountered during use; (3) Suggestions for changes to increase usability; (4) cultural relevance of A4H to their own racial/cultural identity, and (5) Ideas for potential placement of the program. A copy of interview questions are included as appendix to this paper.

## Participants

A total of 32 participants were recruited for usability testing, including Black autistic youth (n = 12), parents and caregivers of Black autistic youth (n = 10), and autism specialists (n = 8). Black autistic youth. Participants were eligible if they identified as Black/African American, were between the ages of 14 and 24, had a 4th-grade reading level or higher, could read and speak English, and had a formal diagnosis of autism documented by medical or school records. Parents were eligible if they had a child or dependent between the ages of 14 and 24 years old, who identified as Black/African American and had a formal diagnosis of autism. Autism specialists were eligible if they had, on average, worked with autistic youth and young adults at least 20 hours per week in roles such as special education teacher, speech pathologist, behavioral therapist, social worker, and so on. Recruitment occurred through community partnerships and professional networks aligned with the study’s focus on mental health equity.

## Ethical and emotional safety safeguards

Given the focus of this study on mental health and depression and the vulnerability of this population, Black autistic youth, it is important to identify the safeguards put in place to ensure participant safety and comfort during their participation in the study. All procedures were approved by the [university name blinded] Institutional Review Board (IRB Protocol Number: 24.034.01-1). Written consent (parents/caregivers) and assent (youth) were obtained prior to participation.

A significant risk is participants reporting being actively suicidal, partaking in self-harm, or reporting a desire to harm others. To mitigate the risk of harm and ensure participant safety, a structured safety protocol was developed and implemented. All participants were required to identify an emergency contact prior to participation and provide their contact information. The PHQ−9 was used for screening and safety monitoring. Scores were reviewed immediately upon completion. Pre-specified thresholds triggered the following protocols: Any response to question 9 of the PHQ-9 (self-harm) greater than 0 activated an immediate risk screen by a trained study clinician, a private check−in with the participant, and, when appropriate, notification to the parent/caregiver or emergency contact, consistent with consent procedures. PHQ-9 scores above 15 (severe depression) prompted a same−day clinical follow−up call and, if needed, a tailored referral for mental health services.

Participants’ use of A4H was conducted live via Zoom with a trained facilitator present throughout. Emotional safety was monitored through: (a) continuous observation for verbal/nonverbal distress cues; (b) pre−specified stop rules (participant request to pause/stop; facilitator concern for escalating distress; disclosure of harm risk); and (c) brief check−ins at midpoint and end (e.g., “How are you doing right now?”; “Would you like to pause?”). If distress was noted, content was paused, grounding strategies were offered, and the session was either modified or terminated at the participant’s preference. The same protocol was used during participant interviews.

Additionally, all participants received a curated resource sheet that included school/community mental health options, culturally responsive providers, and 24/7 crisis lines. Each interview session concluded with a structured debrief to assess emotional state, answer additional questions, review next steps, and provide additional resources if necessary.

## Data analysis

Quantitative survey data were analyzed using descriptive statistics (e.g., means, standard deviations, and frequency distributions) to summarize usability ratings and identify patterns across participant groups. PHQ-9 scores were examined to characterize baseline depressive symptoms within the sample.

Qualitative data from semi−structured interviews were transcribed verbatim and analyzed using a codebook thematic analysis approach (77). We developed a shared codebook anchored deductively to feasibility domains pertinent to this study (acceptability, usability, participant engagement, cultural responsiveness, accessibility) and iteratively refined it inductively as new insights emerged during familiarization and early coding. Two analysts applied the codebook to all transcripts and met regularly to calibrate interpretations, refine code definitions, and promote consistent application across the dataset. Analysts were not blinded to participant group because group membership was evident in the interview context and analytically relevant to our equity−centered aims.

Differences in interpretation were addressed through a multi−step consensus process: paired calibration on the initial transcripts; standing consensus meetings after each coding batch to reconcile discrepancies; iterative codebook revision with version control; and maintenance of an audit trail (decision log and analytic memos capturing code changes, rationales, and theme refinements). When impasses arose, a senior qualitative lead acted as a critical friend to test assumptions and support resolution (78, 79). Themes were generated by grouping related codes into broader patterns aligned with the feasibility aims and were reviewed for coherence and distinctiveness before write−up. Quantitative and qualitative strands were integrated at the interpretation stage (e.g., through matrices linking themes to acceptability/usability indices) to inform iterative refinements to A4H.

### Reflexivity/positionality

Our analytic team (a Black male assistant professor specializing in the mental health of Black autistic youth and an Asian female clinical social worker/youth behavioral therapist) adopted an equity−centered, community−engaged stance. Before coding, brief positionality statements were prepared, along with reflexive memos that described potential lenses and blind spots related to race, disability, and youth help−seeking. Reflexivity continued during coding, codebook revision, and consensus meetings, informing theme development and supporting culturally responsive interpretation for Black autistic youth.

## Findings

### Sample characteristics

Across stakeholder groups, the study engaged Black autistic youth, parents/caregivers, and autism specialists to inform A4H’s design and feasibility. The youth sample (N = 12) had a mean age of 16.7 years and included five females, six males, and one non−binary participant. Two youth identified as biracial (Black and White), and three identified as Haitian American. The parent/caregiver group (N = 10) included eight Black/African American caregivers (three also Haitian American) and two White caregivers; eight were mothers, and two were fathers. The autism specialist group (N = 8) consisted of five White specialists and three Black/African American specialists (one Haitian American). This composition provided culturally relevant perspectives across users and decision−makers who influence youth help−seeking contexts. Demographic data is included in **Table 1**.

**Table 1:** Participant demographics and PHQ-9 results.

Group	N	Age (Mean)	Sex/gender	Race/ethnicity	PHQ-9 Pre (Mean)	PHQ-9 Post (Mean)	SUS (Mean)	Notes
Black autistic youth (AY)	12	16.7	6 male; 5 female; 1 non-binary	2 biracial (Black & White); 3 Haitian American; remainder Black/African American	14.7	14.64	66.2	PHQ-9 collected only for AY
Parents/Caregivers	10	—	8 mothers; 2 fathers	8 Black/African American (3 Haitian American); 2 White	—	—	73.5	PHQ-9 not administered to adults
Autism Specialists	8	—	Not reported	5 White; 3 Black/African American (1 Haitian American)	—	—	71.01	PHQ-9 not administered to adults

PHQ-9 was administered only to Black autistic youth. Mean baseline PHQ-9 = 14.7 (moderate severity); immediate post-test = 14.64 (no short-term change expected given formative design). System Usability Scale (SUS) means: AY = 66, Parents = 73, Specialists = 71.

### Usability and acceptability

Quantitatively, usability varied by stakeholder group. Black autistic youth participants rated A4H’s usability with a mean System Usability Scale (SUS) score of 66.2, slightly below the standard benchmark of 68, an “OK” range that signals overall usability with notable friction points. In contrast, parents/caregivers reported a mean SUS of 73.5 (“Good”), and autism specialists reported 71.01 (“Good”), indicating fewer barriers among adults.

Qualitative feedback from all groups contextualized these differences and pinpointed where youth encountered friction. Youth and adults consistently emphasized the need to simplify language (e.g., replacing terms such as “impaired” or “therapeutic skills” with plain, affirming wording) and to reduce cognitive load. Youth strongly preferred visual learning supports, videos, interactive tutorials, and read−aloud features to scaffold comprehension. Navigation issues (missing back buttons, too many clicks, unclear tutorials) also contributed to cognitive burden. Adults who reported higher SUS scores tended to overlook youth−specific pain points in navigation, micro−copy, and pacing, underscoring the importance of designing first and foremost for the youth experience.

### Symptom severity and short−term change

Youth reported a mean baseline PHQ−9 score of 14.7, indicating moderate depressive symptoms, and no significant short−term change was observed (mean post−test = 14.64). Given A4H’s prototype stage and brief exposure, the absence of symptom change is consistent with a formative usability and feasibility evaluation rather than an efficacy trial. Qualitative data suggest that while A4H provides immediate, structured feedback (a feature youth valued), meaningful symptom reduction likely requires more extended engagement, iteration to reduce usability barriers, and integration into trusted contexts (e.g., schools and community programs).

### Interpersonal safety, trust, and engagement

Interpersonal safety emerged as central to help−seeking. Youth described discomfort initiating help due to fears of burdening caregivers and mental health stigma within Black communities. They valued A4H’s immediate feedback, especially contrasted with delayed responses from adults in real−world settings, and asked for features that normalize help−seeking (e.g., positive reinforcement, strengths−based language, and clear explanations of scores). Requests for confidentiality assurances, non−racist design, and privacy−protective features signal that trust is foundational to sustained engagement.

Virtual characters were pivotal to perceived safety and engagement. Miss Daniels was often perceived as realistic and emotionally responsive. Still, some youth found her intimidating when she appeared distracted or irritable, highlighting the need to calibrate her effect and response clarity. Sage’s visual feedback (thumbs up/down) was appreciated but frequently missed due to its size and placement; youth recommended greater prominence and more explicit signaling. Additionally, adult participants recommended making Sage’s character more youth-presenting so it can be seen as a peer, which would aid participants in navigating the conversation.

### Design features: what worked and what needs change

A4H’s scoring system was motivating for some but discouraging for others when scores were low or insufficiently explained. Youth recommended transparent scoring logic, goal−tracking, and positive micro−reinforcements to sustain motivation. Across groups, participants advocated for shorter, more frequent sessions to maintain attention and reduce fatigue. Navigation changes (e.g., back buttons, streamlined click paths, clear start−here tutorials) were repeatedly cited as essential.

Personalization was widely endorsed: account creation, personalized prompts, and peer forums to foster shared learning and community, paired with robust privacy protections. Participants emphasized multimodal supports (video, interactive demos, read−aloud) and culturally attuned language to reduce cognitive load and enhance accessibility.

### Placement, reach, and integration

Participants identified schools as the most practical setting for A4H, followed by community centers and therapy clinics, where supportive adults and existing routines could scaffold engagement. To extend reach and convenience, participants recommended integrating a mobile app and social media channels that respect privacy while meeting youth where they are. Together, these preferences point to an implementation strategy that blends institutional placement (for visibility and support) with digital touchpoints (for convenience and confidentiality).

Together, the findings indicate that A4H is feasible and generally usable, particularly from adult perspectives, but requires targeted usability and trust enhancements to meet the specific needs of Black autistic youth. Youth−centered changes, such as plain language, multimodal supports, streamlined navigation, transparent and encouraging feedback, emotionally safe virtual agents, and strong privacy assurances, are likely to reduce friction and strengthen engagement. Implementing A4H in schools and community settings, coupled with mobile and social media integration, may further increase access, confidentiality, and sustained use.

## Next steps

The next steps for A4H involve refining the intervention based on comprehensive user feedback to enhance usability, engagement, and cultural responsiveness. Following these modifications, the revised A4H simulation will undergo a mixed-methods evaluation to reassess acceptability, usability, and feasibility. This evaluation will determine whether participants perceive the refined version of A4H as acceptable and usable and whether the intervention can feasibly be delivered at scale. To guide this process, we will apply Yardley and colleagues’ (80) person-based approach, which emphasizes iterative, user-centered design informed by qualitative and quantitative insights into users’ beliefs, attitudes, and contexts (80). This approach ensures that refinements are grounded in the lived experiences of Black autistic youth and their caregivers, making the intervention more relevant, persuasive, and engaging.

Consistent with the iterative user-centered framework previously described, the research team will analyze newly collected data and collaborate closely with the community advisory board to review findings and co-create solutions. Advisory board input will inform decisions about implementing participant feedback and prioritizing changes that improve usability, reliability, and feasibility for Black autistic youth. These steps will position A4H for subsequent efficacy testing, which will evaluate its impact on help-seeking behaviors and depressive symptom disclosure, ultimately preparing the intervention for a full-scale effectiveness trial in school and community settings.

## Planned efficacy evaluation

To evaluate the efficacy of A4H, we will employ a single-group, pretest–posttest quasi-experimental design with approximately 40 Black autistic youth participants. Although a single−group pre–post design is appropriate for establishing preliminary feasibility and acceptability, we acknowledge its inherent limitations, including potential maturation effects, history threats, and the absence of a comparison group (81, 82). These constraints limit causal inference but are acceptable for an early−phase evaluation intended to refine procedures and identify promising signals prior to more rigorous controlled trials. This design is appropriate for an early-stage trial because it allows us to gather preliminary evidence of intervention impact while minimizing resource demands and participant burden. At this stage, the primary goal is to assess whether the refined A4H prototype produces meaningful changes in targeted outcomes under controlled conditions, rather than to establish causal inference. Using a single-group design also enables rapid iteration before committing to the logistical and financial requirements of a randomized controlled trial (RCT).

Additionally, to address limitations inherent in a single−group pre–post design, we incorporated a second post−test assessment to strengthen the interpretability of change over time. Including a follow−up measurement allows us to examine whether observed improvements are sustained beyond the immediate post−intervention period, which helps reduce concerns that changes are solely attributable to short−term reactivity, maturation, or transient exposure effects (83). Although this approach does not fully eliminate threats to internal validity, the addition of a second post−test provides a more robust temporal pattern of outcomes and enhances our ability to distinguish meaningful intervention−related change from short−lived fluctuations. This strategy strengthens the rigor of the early−phase design while maintaining the pragmatic focus appropriate for feasibility testing.

The study will proceed in three phases:

1. Participants will complete a pretest survey assessing baseline knowledge and skills.
2. They will engage with the refined A4H simulation three times over a two-week period, with each session lasting approximately 25-30 minutes.
3. They will complete a posttest survey following the final session.

Additionally, participants will complete a second post-test survey four weeks after the final session to assess retention of knowledge and sustained behavioral intentions. This follow-up helps account for regression toward the mean (83) and provides preliminary evidence of durability. The primary outcomes for the efficacy evaluation will include depression literacy, help-seeking intentions and attitudes, and communication skills for symptom disclosure. Secondary Outcomes will include perceived barriers to care, usability and acceptability of the intervention, and depressive symptom severity. These constructs align with the intervention’s theoretical foundation, which emphasizes the role of knowledge acquisition, self-efficacy enhancement, and behavioral intention formation (in promoting adaptive help-seeking behaviors. Screening, pre-test, and post-test measures are summarized in **Table 2**. Indicators of success will include, (1) significant improvement in depression literacy scores from pretest to posttest, (2) increased help-seeking intention and attitude scores (3) SUS scores ≥ 68 (“OK” usability) and ideally approaching 80 (“Excellent”), (4) positive ratings on acceptability and feasibility measures and, (4) Reduction in perceived barriers to care.

**Table 2:** A4H efficacy evaluation pre- and post-test measures.

Assessment point	Study measures
Screen	• Social Responsivity Scale, 2nd Edition • Demographics
Pre-Test	• Depression Literacy Scale • Patient Health Questionnaire (PHQ-9) • Help-Seeking Intention Scale • Help-Seeking Attitude Scale • General Help-Seeking Questionnaire • Barriers to Access Care Evaluation
Post-Test	• Depression Literacy Scale • Patient Health Questionnaire (PHQ-9) • Help-Seeking Intention Scale • Help-Seeking Attitude Scale • General Help-Seeking Questionnaire • Barriers to Access Care Evaluation • Systems Usability Scale (SUS) • Acceptability of Intervention Measure • Feasibility of Intervention Measure

Measures are organized by assessment point: screening, pre-test, and post-test. PHQ-9, Patient Health Questionnaire-9; SUS, System Usability Scale.

Findings from this phase will inform the design of a future RCT that includes an active control condition and extended follow-up periods (e.g., three to six months) to evaluate sustained effects and mechanisms of change. The RCT will also allow for examination of moderators (e.g., age, autism characteristics) and mediators (e.g., self-efficacy, stigma reduction) to clarify mechanisms of change. Ultimately, these steps will position A4H for a full-scale effectiveness trial and broader implementation in school and community settings.

## Limitations

Although the development and pilot testing of A4H provide promising insights, several limitations should be acknowledged, many of which will be addressed in the subsequent studies described in this manuscript. First, A4H is currently in the prototype stage, which means its features and functionality are still undergoing refinement. As such, findings from this study primarily reflect usability and feasibility rather than efficacy.

Second, the evaluation relied on a small sample size and did not include a control group, limiting the ability to draw causal inferences about intervention effects. As previously stated, the next phase for A4H is an efficacy evaluation that will rely on a single-group pre-test post-test design. This phase will inform a future full-scale evaluation, which will employ a larger, more diverse sample and incorporate randomized controlled designs to strengthen internal validity.

Third, the study design did not include longitudinal follow-up, which is critical for assessing sustained changes in help-seeking behaviors, depression literacy, and mental health outcomes. The described future trial will also incorporate extended observation periods to evaluate the durability of effects and potential long-term benefits.

Finally, while the use of a Community Advisory Board (CAB) enhanced cultural responsiveness and relevance, it may also introduce bias. CAB members’ perspectives, while invaluable, may not fully represent the diversity of experiences within the broader population of Black autistic youth and their families. Future work will triangulate CAB input with broader community engagement strategies and empirical data to mitigate this limitation. Addressing these limitations will be essential for advancing A4H from a promising prototype to a rigorously tested intervention.

## Conclusion: advancing equity through A4H

The development of A4H reflects a deliberate commitment to equity by addressing structural, cultural, and accessibility barriers that disproportionately affect Black autistic youth. Traditional mental health interventions often fail to account for intersecting identities and systemic inequities, leaving marginalized populations underserved. A4H was designed to counter these gaps through multiple equity-driven strategies. A4H employs plain, affirming language and avoids clinical jargon that can alienate youth. Content was iteratively reviewed by a community advisory board to ensure cultural relevance and authenticity, incorporating examples and narratives that resonate with Black autistic youth and their families. Recognizing diverse learning needs, A4H integrates text, audio narration, and visual cues to reduce cognitive load and enhance comprehension. Features such as read-aloud options and interactive tutorials support youth with varying literacy levels and processing styles. To reduce technology-related barriers, A4H is being optimized for mobile delivery. Mobile compatibility ensures that A4H can be accessed in flexible, familiar environments, promoting convenience and scalability. Equity in mental health interventions requires trust. A4H incorporates privacy protections and confidentiality assurances to safeguard user data and foster emotional safety. Virtual simulations provide a secure space to practice help-seeking conversations without fear of stigma or judgment, thereby normalizing disclosure as a proactive and empowering act.

By embedding these equity principles into its design, A4H moves beyond a one-size-fits-all approach to mental health support. It affirms racial and neurodivergent identities, reduces systemic barriers, and promotes culturally grounded pathways to care. Future research will evaluate the extent to which these strategies enhance engagement, usability, and mental health outcomes, positioning A4H as a model for equity-centered innovation in autism and mental health interventions.

## References

[1] Fannin DK Williams E-DG Fuller M Pearson JN Boyd BA Drame ER . Unpacking the prevalence: A warning against overstating the recently narrowed gap for Black autistic youth. Autism Res. (2024) 17:1072–82. doi: 10.1002/aur.3168, PMID: 38804591 PMC11186720

[2] Graf-Kurtulus S Gelo OCG . Rethinking psychological interventions in autism: Toward a neurodiversity-affirming approach. Counselling Psychother Res. (2025) 25:e12874. doi: 10.1002/capr.12874, PMID: 41889077

[3] Magdi HM Abousoliman AD lbrahim AM Elsehrawy MG EL-Gazar HE Zoromba MA . Attention-deficit/hyperactivity disorder and post-traumatic stress disorder adult comorbidity: A systematic review. Systematic Rev. (2025) 14:41. doi: 10.1186/s13643-025-02774-7, PMID: 39953536 PMC11829347

[4] Cage E Cranney R Botha M . Brief report: does autistic community connectedness moderate the relationship between masking and wellbeing? Autism Adulthood. (2022) 4:247–53. doi: 10.1089/aut.2021.0096, PMID: 36606159 PMC9645674

[5] Hotez E Phan JM Truong DM . Addressing stigma-related health disparities for autistic individuals through cultural competemility: insights from research and lived experience. Curr Psychiatry Rep. (2024) 26:761–70. doi: 10.1007/s11920-024-01551-y, PMID: 39460907 PMC11706906

[6] Botha M Gillespie-Lynch K . Come as you are: examining autistic identity development and the neurodiversity movement through an intersectional lens. Hum Dev. (2022) 66:93–112. doi: 10.1159/000524123, PMID: 41869557

[7] Cooper K Russell AJ Lei J Smith LG . The impact of a positive autism identity and autistic community solidarity on social anxiety and mental health in autistic young people. Autism. (2023) 27:848–57. doi: 10.1177/13623613221118351, PMID: 36062470 PMC10074754

[8] Guan S Takahashi F Wada M Takashina HN Ueda M Kawaguchi Y . Understanding autistic identity contingencies: the chain mediation effect of autism acceptance and loneliness in ableist microaggressions and social camouflage. Res Square. (2025) 30(2):466–83. doi: 10.21203/rs.3.rs-5945464/v1, PMID: 41351454 PMC12804416

[9] Rivera RA Bennetto L . Applications of identity-based theories to understand the impact of stigma and camouflaging on mental health outcomes for autistic people. Front Psychiatry. (2023) 14:1243657. doi: 10.3389/fpsyt.2023.1243657, PMID: 37743980 PMC10511883

[10] Choi W-S Woo YS Wang S-M Lim HK Bahk W-M . The prevalence of psychiatric comorbidities in adult ADHD compared with non-ADHD populations: A systematic literature review. PloS One. (2022) 17:e0277175. doi: 10.1371/journal.pone.0277175, PMID: 36331985 PMC9635752

[11] Ware OD Zerden LD Duron JF Xu Y McCarthy LP Verbiest S . Prevalence of co-occurring conditions among youths receiving treatment with primary anxiety, ADHD, or depressive disorder diagnoses. Front Child Adolesc Psychiatry. (2024) 3:1340480. doi: 10.3389/frcha.2024.1340480, PMID: 39816615 PMC11732069

[12] Maenner MJ . Prevalence and characteristics of autism spectrum disorder among children aged 8 years—Autism and developmental disabilities monitoring network, 11 sites, United States 2018, in: MMWR. Surveillance Summaries, Vol. 70 Atlanta, GA USA: Center for Disease Control and Prevention Morbidity and Mortality Weekly Report. (2021). doi: 10.15585/mmwr.ss7011a1, PMID: 34855725 PMC8639024

[13] Shaw KA . Prevalence and early identification of autism spectrum disorder among children aged 4 and 8 years—Autism and developmental disabilities monitoring network, 16 sites, United States 2022, in: MMWR. Surveillance Summaries, Vol. 74 Atlanta, GA USA: Center for Disease Control and Prevention Morbidity and Mortality Weekly Report. (2025). doi: 10.15585/mmwr.ss7402a1, PMID: 40232988 PMC12011386

[14] Leyfer OT Folstein SE Bacalman S Davis NO Dinh E Morgan J . Comorbid psychiatric disorders in children with autism: Interview development and rates of disorders. J Autism Dev Disord. (2006) 36:849–61. doi: 10.1007/s10803-006-0123-0, PMID: 16845581

[15] Menezes M Robinson L Sanchez MJ Cook B . Depression in youth with autism spectrum disorders: A systematic review of studies published between 2012 and 2016. Rev J Autism Dev Disord. (2018) 5:370–89. doi: 10.1007/s40489-018-0146-4, PMID: 41891111

[16] Pezzimenti F Han GT Vasa RA Gotham K . Depression in youth with autism spectrum disorder. Child Adolesc Psychiatr Clinics. (2019) 28:397–409. doi: 10.1016/j.chc.2019.02.009, PMID: 31076116 PMC6512853

[17] Magnuson KM Constantino JN . Characterization of depression in children with autism spectrum disorders. J Dev Behav Pediatrics: JDBP. (2011) 32:332–40. doi: 10.1097/DBP.0b013e318213f56c, PMID: 21502871 PMC3154372

[18] Rai D Culpin I Heuvelman H Magnusson CMK Carpenter P Jones HJ . Association of autistic traits with depression from childhood to age 18 years. JAMA Psychiatry. (2018) 75:835–43. doi: 10.1001/jamapsychiatry.2018.1323, PMID: 29898212 PMC6143081

[19] Hirvikoski T Boman M Chen Q D’Onofrio BM Mittendorfer-Rutz E Lichtenstein P . Individual risk and familial liability for suicide attempt and suicide in autism: A population-based study. psychol Med. (2020) 50:1463–74. doi: 10.1017/S0033291719001405, PMID: 31238998

[20] Santomauro DF Erskine HE Herrera AMM Miller PA Shadid J Hagins H . The global epidemiology and health burden of the autism spectrum: Findings from the Global Burden of Disease Study 2021. Lancet Psychiatry. (2025) 12:111–21. doi: 10.1016/S2215-0366(24)00363-8, PMID: 39709974 PMC11750762

[21] Benevides TW Jaremski JE Williams E-D Song W Pham HH Shea L . Racial and ethnic disparities in community mental health use among autistic adolescents and young adults. J Adolesc Health. (2024) 74(6):1208–16. doi: 10.1016/j.jadohealth.2024.01.036, PMID: 38493400

[22] Kirsch AC Huebner ARS Mehta SQ Howie FR Weaver AL Myers SM . Association of comorbid mood and anxiety disorders with autism spectrum disorder. JAMA Pediatr. (2020) 174:63–70. doi: 10.1001/jamapediatrics.2019.4368, PMID: 31790555 PMC6902186

[23] Assari S Gibbons FX Simons R . Depression among black youth; interaction of class and place. Brain Sci. (2018) 8:6. doi: 10.3390/brainsci8060108, PMID: 29895752 PMC6025590

[24] Liang J Matheson BE Douglas JM . Mental health diagnostic considerations in racial/ethnic minority youth. J Child Family Stud. (2016) 25:1926–40. doi: 10.1007/s10826-015-0351-z, PMID: 27346929 PMC4916917

[25] Opara I Weissinger GM Lardier DT Jr. Lanier Y Carter S Brawner BM . Mental health burden among Black adolescents: The need for better assessment, diagnosis and treatment engagement. Soc Work Ment Health. (2021) 19:88–104. doi: 10.1080/15332985.2021.1879345, PMID: 34248423 PMC8262091

[26] Bernard DL Smith Q Lanier P . Racial discrimination and other adverse childhood experiences as risk factors for internalizing mental health concerns among Black youth. J Traumatic Stress. (2022) 35:473–83. doi: 10.1002/jts.22760, PMID: 34800051 PMC9035019

[27] Lavner JA Hart AR Carter SE Beach SRH . Longitudinal effects of racial discrimination on depressive symptoms among black youth: between- and within-person effects. J Am Acad Child Adolesc Psychiatry. (2022) 61:56–65. doi: 10.1016/j.jaac.2021.04.020, PMID: 34015482 PMC8599529

[28] Henderson C Evans-Lacko S Thornicroft G . Mental illness stigma, help seeking, and public health programs. Am J Public Health. (2013) 103:777–80. doi: 10.2105/AJPH.2012.301056, PMID: 23488489 PMC3698814

[29] Lindsey MA Joe S Nebbitt V . Family matters: the role of mental health stigma and social support on depressive symptoms and subsequent help seeking among African American boys. J Black Psychol. (2010) 36:458–82. doi: 10.1177/0095798409355796, PMID: 20953336 PMC2953262

[30] Davis AM Smith E Yang X Wright R . Exploring racial discrimination, disability discrimination, and perception of the future among black-identifying emerging adults with and without autism in the United States: A mixed-methods descriptive study. J Child Adolesc Trauma. (2024) 17(4):1019–34. doi: 10.1007/s40653-024-00624-7, PMID: 39686937 PMC11646248

[31] Williams E-DG Smith MJ Sherwood K Lovelace TS Bishop L . Brief report: initial evidence of depressive symptom disparities among black and white transition age autistic youth. J Autism Dev Disord. (2022) 52:3740–5. doi: 10.1007/s10803-021-05242-y, PMID: 34417653 PMC8858325

[32] Davis A Solomon M Belcher H . Examination of race and autism intersectionality among African American/Black young adults. Autism Adulthood: Challenges Manage. (2022) 4:306–14. doi: 10.1089/aut.2021.0091, PMID: 36777378 PMC9908282

[33] Roux AM Voltaire S Steinberg H Williams ED Anderson KA Hutson TM . More Than Just a Variable: The Need to Explicitly Focus on Black Youth Within Autism Transitions Research. Autism Adulthood. (2024) 6:119–127. doi: 10.1089/aut.2023.0041, PMID: 39144071 PMC11320561

[34] Golson ME Ficklin E Haverkamp CR McClain MB Harris B . Cultural differences in social communication and interaction: A gap in autism research. Autism Res. (2022) 15:208–14. doi: 10.1002/aur.2657, PMID: 34936220

[35] Jones CD Schwartz IS . When asking questions is not enough: an observational study of social communication differences in high functioning children with autism. J Autism Dev Disord. (2009) 39:432–43. doi: 10.1007/s10803-008-0642-y, PMID: 18784993

[36] Banks BM Jackson T Goodwin R Moore R . Autism is black too! Intersectional experiences with service provision for black autistic individuals and their families. J Soc Issues. (2025) 81:e70024. doi: 10.1111/josi.70024, PMID: 41875165

[37] Yang X Wu J Ma Y Yu J Cao H Zeng A . Effectiveness of virtual reality technology interventions in improving the social skills of children and adolescents with autism: systematic review. J Med Internet Res. (2025) 27:e60845. doi: 10.2196/60845, PMID: 39907288 PMC11840372

[38] Zhao J Zhang X Lu Y Wu X Zhou F Yang S . Virtual reality technology enhances the cognitive and social communication of children with autism spectrum disorder. Front Public Health. (2022) 10:2022.1029392. doi: 10.3389/fpubh.2022.1029392, PMID: 36276341 PMC9582941

[39] Williams B Reddy P Marshall S Beovich B McKarney L . Simulation and mental health outcomes: A scoping review. Adv Simulation. (2017) 2:2. doi: 10.1186/s41077-016-0035-9, PMID: 29450003 PMC5806484

[40] Jones JM . Culturally responsive adaptations to evidence-based interventions for Black adolescents. School Psychol. (2025) 40:274–85. doi: 10.1037/spq0000688, PMID: 40193509

[41] Kalibatseva Z Leong FTL . A critical review of culturally sensitive treatments for depression: Recommendations for intervention and research. psychol Serv. (2014) 11:433–50. doi: 10.1037/a0036047, PMID: 25383996

[42] Lee JD Kang VY Terol AK Joo S . Examining the efficacy of culturally responsive interventions for autistic children and their families: A meta-analysis. J Autism Dev Disord. (2025) 55:706–26. doi: 10.1007/s10803-023-06212-2, PMID: 38246962 PMC11260274

[43] Pham AV Charles LC . Racial disparities in autism diagnosis, assessment, and intervention among minoritized youth: sociocultural issues, factors, and context. Curr Psychiatry Rep. (2023) 25:201–11. doi: 10.1007/s11920-023-01417-9, PMID: 37004631

[44] Pearson JN Stewart-Ginsburg JH Malone K Manns L Mason Martin D Sturdivant D . Best FACES forward: outcomes of an advocacy intervention for black parents raising autistic youth. Exceptionality. (2023) 31:135–48. doi: 10.1080/09362835.2022.2100392, PMID: 41891072

[45] Annamma SA Connor D Ferri B . Dis/ability critical race studies (DisCrit): Theorizing at the intersections of race and dis/ability. Race Ethnicity Educ. (2013) 16:1–31. doi: 10.1080/13613324.2012.730511, PMID: 41891072

[46] Uzoaru F Oleribe OO Collins L Preston M Ross L Chadha D . Addressing racial inequities: A systematic review of intervention programs for Black/African American children with autism spectrum disorder and attention-deficit hyperactivity disorder (2019–2024). Pediatr Med. (2025) 8. doi: 10.21037/pm-24-75, PMID: 41835801

[47] Machalicek W Glugatch L Erturk B Brafford T Kunze M Drew C . Recommendations for diversifying racial and ethnic representation in autism intervention research: A crossover review of recruitment and retention practices in pediatric mental health. J Clin Med. (2022) 11:6468. doi: 10.3390/jcm11216468, PMID: 36362698 PMC9654487

[48] Williams EDG Smith MJ Mitchell J Tucker TB Sung C Sherwood K . The development and utilization of a diversity advisory board in an intervention to support social skill development for autistic transition-aged youth. Autism. (2025). Available online at: https://journals.sagepub.com/eprint/GTPBD6RSD9Z3Q7W4RVUJ/full. 10.1177/13623613251330847 PMC13463652 40231706

[49] Bitsika V Sharpley CF . The Association Between Social Responsivity and Depression in High-Functioning Boys with an Autism Spectrum Disorder. J Dev Phys Disabil. (2016) 28:317–331. doi: 10.1007/s10882-015-9470-0, PMID: 41891111

[50] Walton QL Payne JS . Missing the Mark: Cultural Expressions of Depressive Symptoms Among African American Women and Men. Internet. (2024). Available online at: https://www.academia.edu/21595399/Missing_the_Mark_Cultural_Expressions_of_Depressive_Symptoms_Among_African_American_Women_and_Men.

[51] Cauce AM Domenech-Rodríguez M Paradise M Cochran BN Shea JM Srebnik D . Cultural and contextual influences in mental health help seeking: A focus on ethnic minority youth. J Consulting Clin Psychol. (2002) 70:44–55. doi: 10.1037/0022-006X.70.1.44, PMID: 11860055

[52] Barkin S Schlundt D Smith P . Community-engaged research perspectives: then and now. Acad Pediatr. (2013) 13:93–7. doi: 10.1016/j.acap.2012.12.006, PMID: 23498079

[53] Luger TM Hamilton AB True G . Measuring community-engaged research contexts, processes, and outcomes: A mapping review. Milbank Q. (2020) 98:493–553. doi: 10.1111/1468-0009.12458, PMID: 32428339 PMC7296434

[54] Abras C Maloney-Krichmar D Preece J . User-centered design. In: Bainbridge W , editor. Encyclopedia of Human-Computer Interaction, vol. 37 . Sage Publications, Thousand Oaks (2004). p. 445–56.

[55] Sanders E . From user-centered to participatory design approaches. In: Design and the Social Sciences: Making Connections. London: CRC Press (2002). p. 1–7. doi: 10.1201/9780203301302.ch1

[56] Clinical and Translational Science Awards Consortium Community Engagement Key Function Committee Task Force on the Principles of Community Engagement, Services, U. States D. @ of H. and H., National Institutes of Health (U.S.), Centers for Disease Control and Prevention (U.S.), & United States Agency for Toxic Substances and Disease Registry . Principles of community engagement. 2nd ed. Bethesda Maryland: Dept. of Health & Human Services, National Institutes of Health, Centers for Disease Control and Prevention, Agency for Toxic Substances and Disease Registry, Clinical and Translational Science Awards (2011).

[57] Fortuna K Barr P Goldstein C Walker R Brewer L Zagaria A . Application of community-engaged research to inform the development and implementation of a peer-delivered mobile health intervention for adults with serious mental illness. J Participatory Med. (2019) 11:e12380. doi: 10.2196/12380, PMID: 32095314 PMC7039401

[58] Beres LK Simbeza S Holmes CB Mwamba C Mukamba N Sharma A . Human-centered design lessons for implementation science: improving the implementation of a patient-centered care intervention. JAIDS J Acquired Immune Deficiency Syndromes. (2019) 82:S230. doi: 10.1097/QAI.0000000000002216, PMID: 31764259 PMC6880397

[59] Lyon AR Koerner K . User-centered design for psychosocial intervention development and implementation. Clin Psychology: Sci Pract. (2016) 23:180–200. doi: 10.1111/cpsp.12154, PMID: 29456295 PMC5812700

[60] Vial S Boudhraâ S Dumont M . Human-centered design approaches in digital mental health interventions: exploratory mapping review. JMIR Ment Health. (2022) 9:e35591. doi: 10.2196/35591, PMID: 35671081 PMC9214621

[61] International Organization for Standardization . *ISO 9241-210:2019—Ergonomics of human-system interaction—Part 210: Human-centred design for interactive systems* (No. 2). Geneva, Switzerland: International Organization for Standardization (2019). Available online at: https://webstore.ansi.org/standards/iso/iso92412102019?ad_acct=0000&gad_source=1&gad_campaignid=1042027569&gbraid=0AAAAAD_gXFUQj3Dtp3iegdcjwjcyxQwNp&gclid=Cj0KCQiArt_JBhCTARIsADQZayk8hnf2M50gZBHfdT1vHn2GPzHhpyu_VlnTK1AR3gcnjge16JoBqqwaAnC6EALw_wcB (Accessed January 12, 2026).

[62] Furnham A Swami V . Mental health literacy: A review of what it is and why it matters. Int Perspect Psychol. (2018) 7:240–57. doi: 10.1037/ipp0000094, PMID: 41770175

[63] Jorm AF . Mental health literacy. Public knowledge and beliefs about mental disorders. Br J Psychiatry: J Ment Sci. (2000) 177:396–401. doi: 10.1192/bjp.177.5.396, PMID: 11059991

[64] Jorm AF Korten AE Jacomb PA Christensen H Rodgers B Pollitt P . Mental health literacy”: A survey of the public’s ability to recognise mental disorders and their beliefs about the effectiveness of treatment. Med J Aust. (1997) 166:182–6. doi: 10.5694/j.1326-5377.1997.tb140071.x, PMID: 9066546

[65] Annamma SA Ferri BA Connor DJ . Disability critical race theory: exploring the intersectional lineage, emergence, and potential futures of DisCrit in education. Rev Res Educ. (2018) 42:46–71. doi: 10.3102/0091732X18759041, PMID: 38293548

[66] Lindsey MA Mufson L Vélez-Grau C Grogan T Wilson DM Reliford AO . Engaging Black youth in depression and suicide prevention treatment within urban schools: Study protocol for a randomized controlled pilot. Trials. (2024) 25:112. doi: 10.1186/s13063-024-07947-8, PMID: 38336803 PMC10854091

[67] Lanzieri N Dempsey A Olsen AF Samelson H Plass JL . Creating an AI powered VR simulation platform for social work skill development. J Technol Hum Serv. (2025) 0:1–29. doi: 10.1080/15228835.2025.2565820, PMID: 41891072

[68] Waller BY Joyce PA Quinn CR Hassan Shaari AA Boyd DT . “ I am the one that needs help”: the theory of help-seeking behavior for survivors of intimate partner violence. J Interpers Violence. (2023) 38:288–310. doi: 10.1177/08862605221084340, PMID: 35350920 PMC9519802

[69] Ajzen I . The theory of planned behavior. Organizational Behav Hum Decision Processes. (1991) 50:179–211. doi: 10.1016/0749-5978(91)90020-T

[70] Bosnjak M Ajzen I Schmidt P . The Theory of Planned Behavior: Selected Recent Advances and Applications. Eur J Psychol. (2020) 16:352–356. doi: 10.5964/ejop.v16i3.3107, PMID: 33680187 PMC7909498

[71] Adams C Gringart E Strobel N . Explaining adults’ mental health help-seeking through the lens of the theory of planned behavior: A scoping review. Systematic Rev. (2022) 11:160. doi: 10.1186/s13643-022-02034-y, PMID: 35945633 PMC9361557

[72] Hammer JH Vogel DL Grzanka PR Kim N Keum BT Adams C . The integrated behavioral model of mental health help seeking (IBM-HS): A health services utilization theory of planned behavior for accessing care. J Couns Psychol. (2024) 71:315–27. doi: 10.1037/cou0000754, PMID: 39115906

[73] Bandura A . Self-Efficacy: the foundation of agency1. In: Control of Human Behavior, Mental Processes, and Consciousness. Mahwah New Jersey: Psychology Press (2000).

[74] Rickwood D Deane FP Wilson CJ Ciarrochi J . Young people’s help-seeking for mental health problems. Aust E-Journal Advancement Ment Health. (2005) 4:218–51. doi: 10.5172/jamh.4.3.218

[75] Arnold SRC Uljarević M Hwang YI Richdale AL Trollor JN Lawson LP . Brief report: psychometric properties of the patient health questionaire-9 (PHQ-9) in autistic adults. J Autism Dev Disord. (2020) 50:2217–25. doi: 10.1007/s10803-019-03947-9, PMID: 30847710

[76] Mol M van Schaik A Dozeman E Ruwaard J Vis C Ebert DD . Dimensionality of the system usability scale among professionals using internet-based interventions for depression: A confirmatory factor analysis. BMC Psychiatry. (2020) 20:218. doi: 10.1186/s12888-020-02627-8, PMID: 32398111 PMC7216472

[77] Braun V Clarke V . Thematic analysis. In: APA Handbook of Research Methods in Psychology, 2nd ed, vol. 2. Washington, DC: American Psychological Association (2023). p. 65–81. doi: 10.1037/0000319-004, PMID: 41770175

[78] Braun V Clarke V . A critical review of the reporting of reflexive thematic analysis in Health Promotion International. Health Promotion Int. (2024) 39:daae049. doi: 10.1093/heapro/daae049, PMID: 38805676 PMC11132294

[79] Giesen L Roeser A . Structuring a team-based approach to coding qualitative data. Int J Qual Methods. (2020) 19:160940692096870. doi: 10.1177/1609406920968700, PMID: 41883860

[80] Yardley L Ainsworth B Arden-Close E Muller I . The person-based approach to enhancing the acceptability and feasibility of interventions. Pilot Feasibility Stud. (2015) 1:37. doi: 10.1186/s40814-015-0033-z, PMID: 27965815 PMC5153673

[81] Spurlock DR . The Single-Group, Pre- and Posttest Design in Nursing Education Research: It’s Time to Move on. J Nurs Educ. (2018) 57:69–71. doi: 10.3928/01484834-20180123-02, PMID: 29384566

[82] Bierer SB Beck DG Borges NJ Brondfield S Fung CC Huggett KN . Moving Beyond Simplistic Research Design in Health Professions Education: What a One-Group Pretest-Posttest Design Will Not Prove. MedEdPORTAL. (2025) 21:11527. doi: 10.15766/mep_2374-8265.11527, PMID: 40395408 PMC12089416

[83] Marsden E Torgerson CJ . Single group, pre- and post-test research designs: Some methodological concerns. Oxf Rev Educ. (2012) 38:583–616. doi: 10.1080/03054985.2012.731208, PMID: 41891072

